# Severe First-Trimester Megacystis (>15 mm): A Case Report of Prognostic and Counseling Challenges Without Invasive Testing

**DOI:** 10.7759/cureus.105142

**Published:** 2026-03-12

**Authors:** Debabrata Maitra, Ansuman Sarkar

**Affiliations:** 1 Radiology, College of Medicine, Sagore Dutta Hospital, Kolkata, IND; 2 Obstetrics and Gynecology, Basirhat District Hospital, Basirhat, IND

**Keywords:** fetal bladder distension, first-trimester megacystis, lower urinary tract obstruction, prenatal ultrasound, prognostic counseling

## Abstract

First-trimester fetal megacystis is an uncommon but clinically significant finding on prenatal ultrasound because of its association with chromosomal anomalies and lower urinary tract obstruction. A 32-year-old gravida 2 para 1 woman presented for routine first-trimester evaluation at 12+6 weeks’ gestation. Sonography revealed a viable fetus with a crown-rump length of 64.3 mm and a fetal heart rate of 175 bpm. A grossly distended urinary bladder measuring 19.4 × 11.0 mm was noted. Nuchal translucency, nasal bone, and ductus venosus Doppler studies were normal. No keyhole sign or changes in renal echogenicity were observed. Amniotic fluid volume and placental morphology were consistent with gestational age. After thorough counseling about the risks of chromosomal abnormalities and progressive obstructive uropathy, the family refused invasive procedures and chose surgical termination. Histopathological evaluation was not performed. Severe first-trimester megacystis is strongly associated with adverse perinatal outcomes. This case highlights the prognostic uncertainty and counseling challenges encountered when invasive diagnostic confirmation is unavailable.

## Introduction

Fetal megacystis is defined as an enlarged fetal urinary bladder detected on prenatal ultrasound, typically characterized by a longitudinal bladder diameter greater than 7 mm during the first trimester of pregnancy [1]. Although it is a relatively rare condition, early detection is clinically important because it may be associated with chromosomal abnormalities, particularly trisomy 13 and trisomy 18, as well as structural anomalies such as lower urinary tract obstruction (LUTO) [2].

The prognosis of fetal megacystis largely depends on the degree of bladder enlargement. Studies have shown that when the longitudinal bladder diameter is less than 15 mm, spontaneous resolution may occur in a significant proportion of cases, whereas measurements greater than 15 mm are associated with an increased risk of aneuploidy, urinary tract obstruction, renal impairment, and adverse perinatal outcomes [3]. Therefore, first-trimester ultrasound screening between 11 and 13+6 weeks of gestation plays an important role in early detection and risk stratification [4].

Recent advances in prenatal imaging and prenatal genetic testing have further improved the evaluation and prognostic assessment of fetuses with megacystis and other structural abnormalities detected during early pregnancy [5-7].

## Case presentation

A 32-year-old gravida 2 para 1 patient presented for routine first-trimester screening at 12+6 weeks of gestation. There were no maternal comorbidities, consanguinity, or identifiable risk factors. Transabdominal ultrasonography was performed using a GE Versana Balance R2 VA system (GE Medical Systems Co., Ltd., Wuxi, China) with a 4C-RS convex transducer (GE Medical Systems Co., Ltd.).

**Figure 1:**
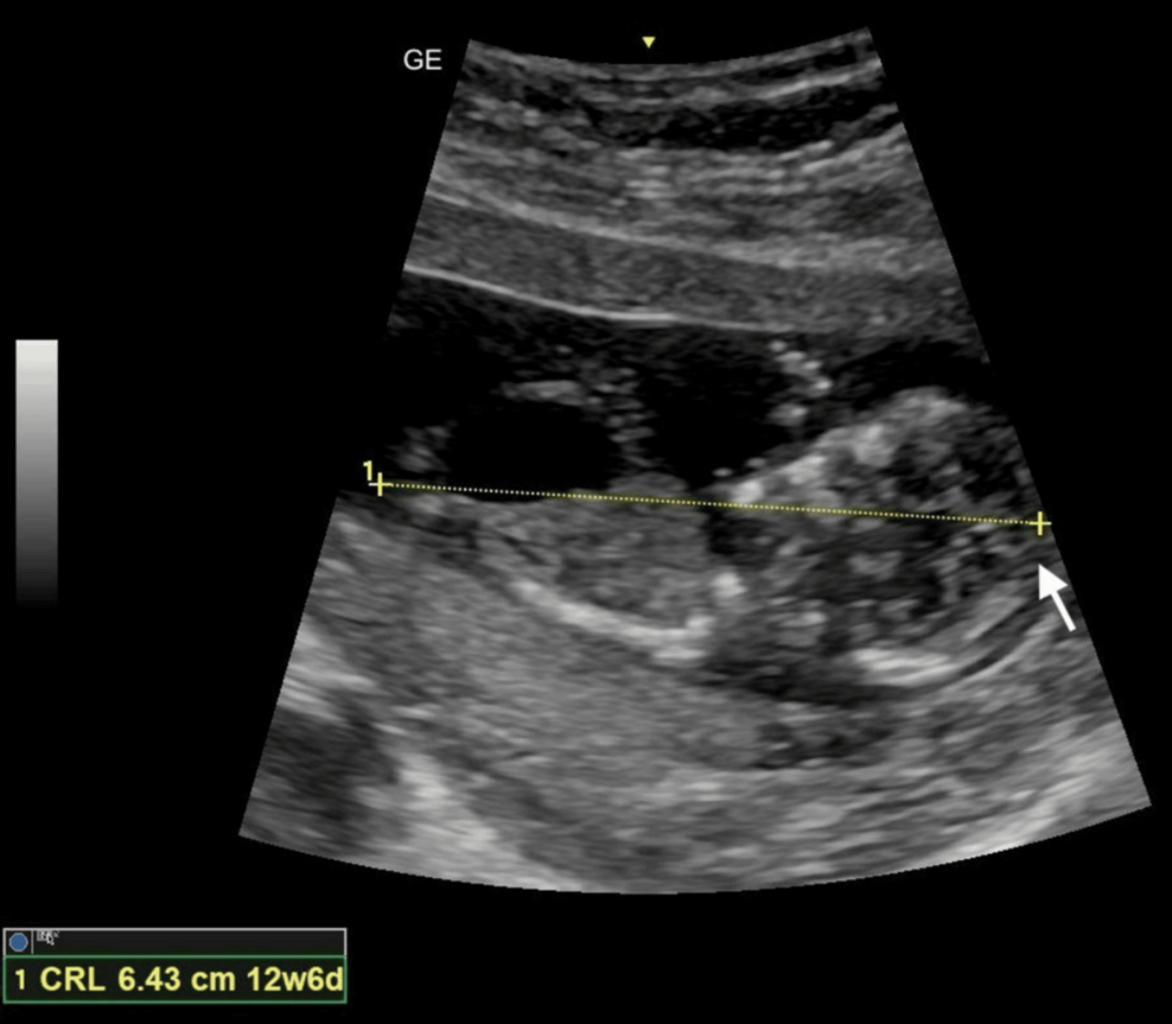
Grayscale transabdominal ultrasound image demonstrating a crown-rump length of 64.3 mm (the arrow indicates the fetal crown)

**Figure 2:**
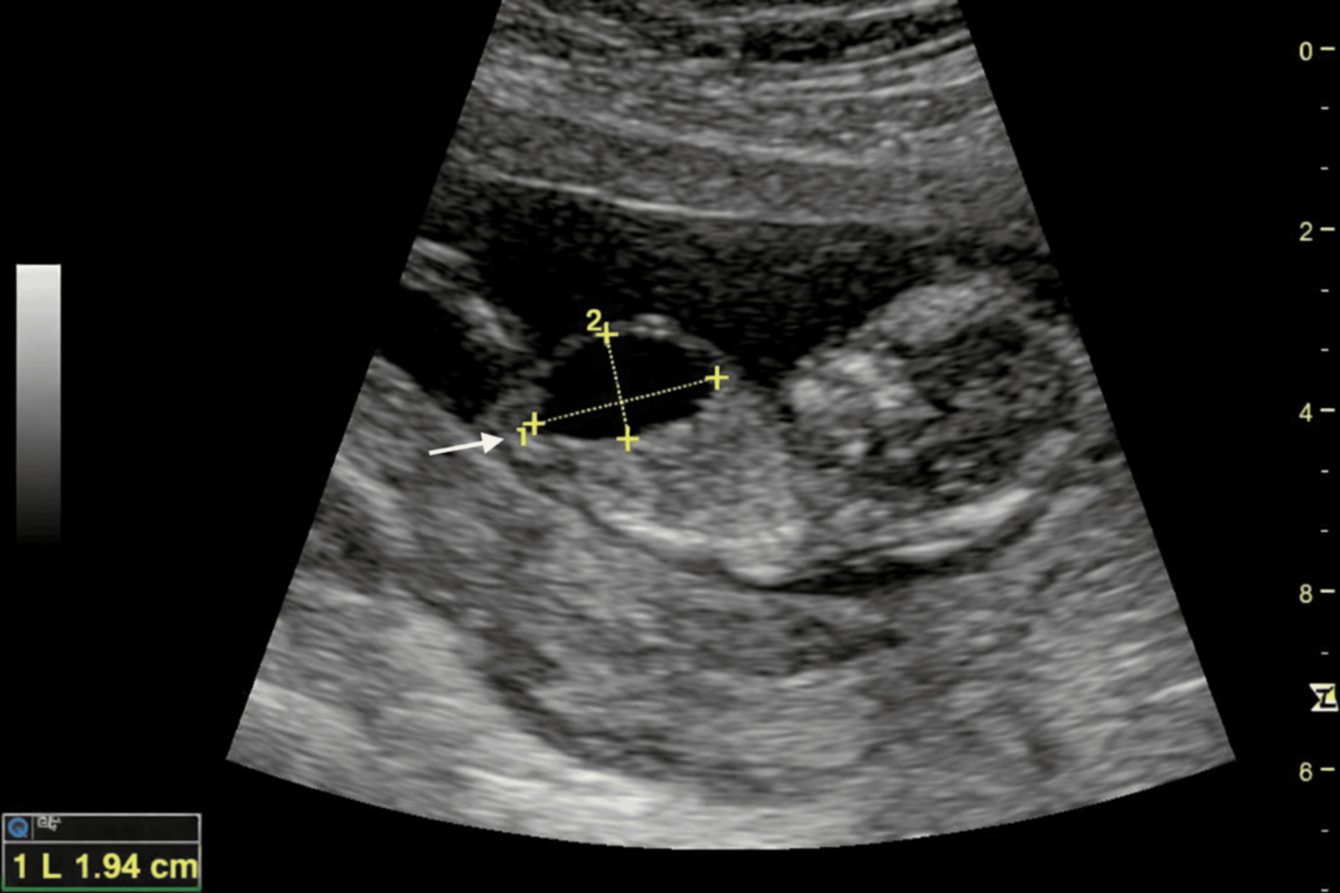
Grayscale transabdominal ultrasound image at 12+6 weeks’ gestation showing a markedly distended fetal urinary bladder with a longitudinal diameter of 19.4 mm (arrow indicates the distended urinary bladder)

**Figure 3:**
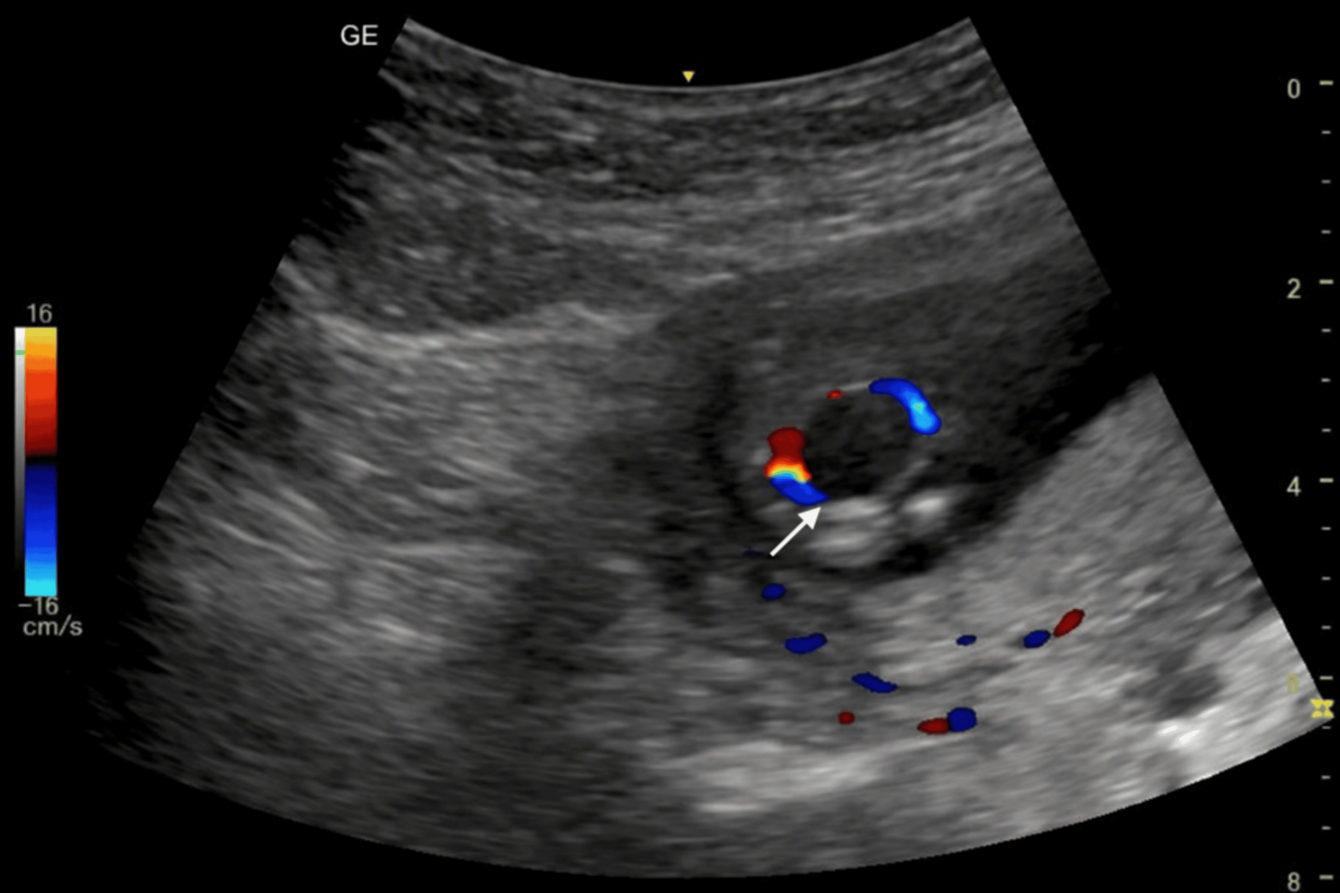
Color Doppler ultrasound image demonstrating visualization of both umbilical arteries adjacent to the distended bladder (arrow indicates the urinary bladder). No “keyhole sign” is seen

The examination demonstrated a crown-rump length of 64.3 mm consistent with gestational age (Figure 1) and a fetal heart rate of 175 bpm. A markedly distended urinary bladder measuring approximately 19.4 × 11.0 mm was observed, consistent with fetal megacystis (Figure 2). Color Doppler imaging demonstrated the presence of both umbilical arteries coursing on either side of the bladder (Figure 3).

**Figure 4:**
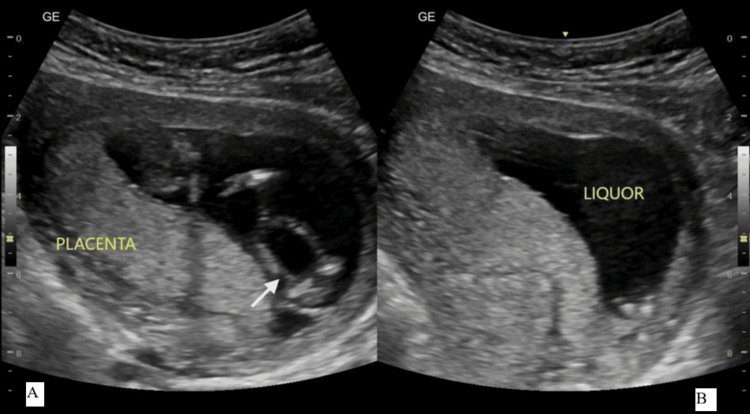
(A) Grayscale transabdominal ultrasound image at 12+6 weeks showing persistent bladder enlargement (arrow indicates the urinary bladder). (B) Image demonstrating adequate amniotic fluid volume and normal placental appearance

**Figure 5:**
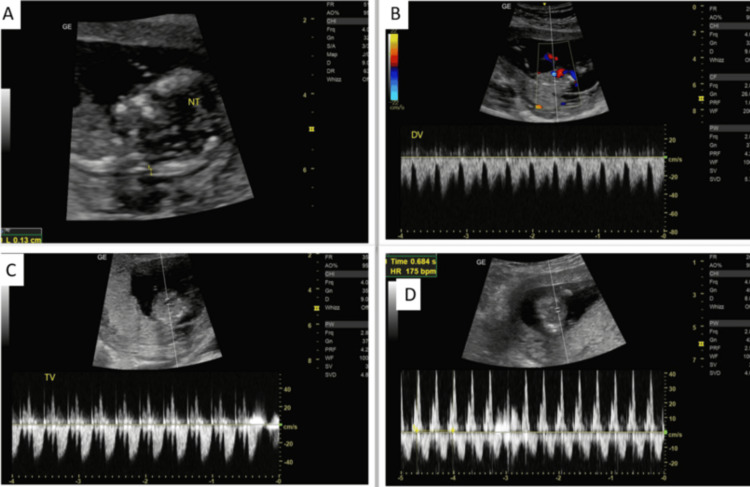
(A) Ultrasound image demonstrating measurement of nuchal translucency. (B) Color Doppler and spectral Doppler evaluation of the ductus venosus demonstrating a normal waveform. (C) Tricuspid valve Doppler assessment demonstrating absence of tricuspid regurgitation. (D) Spectral Doppler trace demonstrating fetal heart rate measurement (175 bpm)

Further evaluation demonstrated normal amniotic fluid volume and normal placental morphology for gestational age (Figure 4). First-trimester screening parameters were also assessed. The nuchal translucency measured 1.3 mm, the nasal bone was present, measuring 2.1 mm, and the ductus venosus Doppler waveform was normal. Tricuspid valve Doppler assessment showed no evidence of tricuspid regurgitation, and spectral Doppler demonstrated fetal heart rate measurement (Figure 5).

No keyhole sign was observed, and there was no evidence of abnormal renal echogenicity. As per national regulations prohibiting prenatal sex determination, the sex of the fetus was not evaluated.

Further diagnostic investigations, including chorionic villus sampling and amniocentesis, were offered but declined by the family after counseling. The differential diagnoses included LUTO, such as posterior urethral valves or urethral atresia; chromosomal abnormalities, including trisomy 13 and trisomy 18; and isolated or transient megacystis.

After detailed counseling regarding the possible risks of chromosomal abnormalities, progressive renal impairment, pulmonary hypoplasia, and adverse perinatal outcomes, the family opted for termination of pregnancy. Surgical termination was performed at 13 weeks of gestation without maternal complications. Histopathological examination was not performed due to financial constraints; therefore, a definitive etiological diagnosis could not be established.

## Discussion

Fetal megacystis detected during the first trimester is an uncommon but clinically significant finding on prenatal ultrasound. It is typically defined as a longitudinal bladder diameter greater than 7 mm between 10 and 14 weeks of gestation [1]. Early identification during routine first-trimester screening is important because fetal megacystis may be associated with chromosomal abnormalities, congenital anomalies, and LUTO [2].

The prognosis of fetal megacystis largely depends on the degree of bladder enlargement and the presence of associated abnormalities. Previous studies have demonstrated that when the longitudinal bladder diameter is less than 15 mm, spontaneous resolution may occur in a considerable proportion of cases. In contrast, larger measurements are associated with an increased likelihood of persistent obstruction, renal impairment, and adverse pregnancy outcomes [3]. Consequently, bladder size is an important prognostic indicator in early pregnancy.

The differential diagnosis of first-trimester megacystis includes obstructive conditions such as posterior urethral valves and urethral atresia, as well as chromosomal abnormalities and syndromic conditions. Chromosomal anomalies, particularly trisomy 13 and trisomy 18, have been reported in association with fetal megacystis, highlighting the importance of appropriate genetic counseling and prenatal diagnostic evaluation [2].

Advances in prenatal imaging have improved the early evaluation of fetuses with suspected urinary tract abnormalities. Comprehensive first-trimester ultrasound assessment typically includes evaluation of nuchal translucency, ductus venosus Doppler flow, tricuspid valve Doppler assessment, and a detailed anatomical survey. These parameters may help identify associated abnormalities and contribute to improved risk stratification [4].

Recent studies evaluating fetal megacystis diagnosed in the first trimester have further emphasized the prognostic importance of bladder size and associated anomalies in predicting pregnancy outcome [5,6]. More recent analyses have also compared outcomes between different longitudinal bladder diameter groups to improve prenatal counseling and clinical decision-making [7].

In the present case, the bladder measurement of approximately 19.4 mm placed the fetus in a high-risk category associated with poor prognosis. Although classic sonographic signs of severe obstruction, such as the keyhole sign, were not observed and renal echogenicity appeared normal, evolving LUTO could not be excluded. The absence of invasive genetic testing and posttermination pathological evaluation limited the ability to determine the precise underlying etiology.

This case highlights the diagnostic and counseling challenges encountered when fetal megacystis is detected during the first trimester and invasive testing is declined. Careful interpretation of ultrasound findings and thorough counseling regarding possible outcomes remain essential for guiding clinical decision-making.

## Conclusions

First-trimester fetal megacystis is an important prenatal ultrasound finding that may be associated with chromosomal abnormalities and LUTO. Early detection during routine first-trimester screening allows appropriate risk assessment and counseling regarding potential pregnancy outcomes. This case highlights the importance of careful ultrasound evaluation and individualized counseling when fetal megacystis is identified, particularly when invasive prenatal diagnostic testing is declined.
